# On the Role of Aggregation Prone Regions in Protein Evolution, Stability, and Enzymatic Catalysis: Insights from Diverse Analyses

**DOI:** 10.1371/journal.pcbi.1003291

**Published:** 2013-10-17

**Authors:** Patrick M. Buck, Sandeep Kumar, Satish K. Singh

**Affiliations:** Pharmaceutical Research and Development, Biotherapeutics Pharmaceutical Sciences, Pfizer Inc., Chesterfield, Missouri, United States of America; University of California San Diego, United States of America

## Abstract

The various roles that aggregation prone regions (APRs) are capable of playing in proteins are investigated here *via* comprehensive analyses of multiple non-redundant datasets containing randomly generated amino acid sequences, monomeric proteins, intrinsically disordered proteins (IDPs) and catalytic residues. Results from this study indicate that the aggregation propensities of monomeric protein sequences have been minimized compared to random sequences with uniform and natural amino acid compositions, as observed by a lower average aggregation propensity and fewer APRs that are shorter in length and more often punctuated by gate-keeper residues. However, evidence for evolutionary selective pressure to disrupt these sequence regions among homologous proteins is inconsistent. APRs are less conserved than average sequence identity among closely related homologues (≥80% sequence identity with a parent) but APRs are more conserved than average sequence identity among homologues that have at least 50% sequence identity with a parent. Structural analyses of APRs indicate that APRs are three times more likely to contain ordered versus disordered residues and that APRs frequently contribute more towards stabilizing proteins than equal length segments from the same protein. Catalytic residues and APRs were also found to be in structural contact significantly more often than expected by random chance. Our findings suggest that proteins have evolved by optimizing their risk of aggregation for cellular environments by both minimizing aggregation prone regions and by conserving those that are important for folding and function. In many cases, these sequence optimizations are insufficient to develop recombinant proteins into commercial products. Rational design strategies aimed at improving protein solubility for biotechnological purposes should carefully evaluate the contributions made by candidate APRs, targeted for disruption, towards protein structure and activity.

## Author Summary

Biotechnology requires the large-scale expression, yield, and storage of recombinant proteins. Each step in protein production has the potential to cause aggregation as proteins, not evolved to exist outside the cell, endure the various steps involved in commercial manufacturing processes. Mechanistic studies into protein aggregation have revealed that certain sequence regions contribute more to the aggregation propensity of a protein than other sequence regions do. Efforts to disrupt these regions have thus far indicated that rational sequence engineering is a useful technique to reduce the aggregation of biotechnologically relevant proteins. To improve our ability to rationally engineer proteins with enhanced expression, solubility, and shelf-life we conducted extensive analyses of aggregation prone regions (APRs) within protein sequences to characterize the various roles these regions play in proteins. Findings from this work indicate that protein sequences have evolved by minimizing their aggregation propensities. However, we also found that many APRs are conserved in protein families and are essential to maintain protein stability and function. Therefore, the contributions that APRs, targeted for disruption, make towards protein stability and function should be carefully evaluated when improving protein solubility *via* rational design.

## Introduction

Irreversible β-strand driven protein aggregation and amyloidogenesis is a tremendous burden to biological organisms. Protein loss-of function due to aggregation causes stress to the cell and metabolic energy is lost on the expression, synthesis, and degradation of proteins which aggregate. To overcome these challenges and build cellular machineries that can sustain metabolic flux, higher organisms have developed sophisticated protein quality control mechanisms, including molecular chaperones, post-translational modifications, and degradation/clearance pathways to prevent aggregation from disrupting homeostasis [1]–[3]. When quality control mechanisms are impaired, due to aging or otherwise, protein aggregation can lead to ‘conformational diseases’ in humans and animals [1], [3]–[5].

Despite its deleterious effects, protein aggregation remains unavoidable due to the inherent physico-chemical properties of protein sequences and the formation of non-native conformations due to sequence mutation or unfolding events in response to environmental stress. However, studies of amyloidogenic proteins have revealed that different protein sequences vary in their propensity to aggregate, which can be attributed to the presence of aggregation-nucleating short sequence stretches, capable of forming the cross-β steric zipper motif, called aggregation prone regions (APRs) [6]–[10]. Analyses of APRs indicate common sequence properties including a high preference for β-branched hydrophobic residues, strong β-sheet propensity, low net charge, and in the case of fibril forming patterns, position-specific charged residues [11], [12]. Knowledge of these properties has enabled the development of phenomenological and first-principle based methods to predict APRs in any protein sequence [13]–[20].

The availability of computational APR prediction tools has facilitated large-scale investigations into the aggregation propensities of protein sequences [21]–[27]. Analyzing intrinsically disordered protein (IDP) sequences using APR prediction tools has revealed that the number of APRs found in IDPs is three times less than those found in sequences for ordered proteins [21]. Given the tendency for APRs to exist in ordered sequence regions, it was proposed that APRs may have a role in promoting structural order in native folds. More recent studies have extended the concept that APRs can play a role in promoting structural order based on the prevalence of APRs in protein-protein interactions sites [22], [28], including antibody-antigen interfaces [23]. In fact, the trend for APRs to exist in protein-protein interaction sites has led some research groups to repurpose their APR prediction methods into tools for identifying potential protein-ligand [29] and protein-protein interaction sites [28]. On the other hand, mounting evidence from analyses of large sequence datasets strongly suggests that nature is actively minimizing the occurrence/impact of aggregation prone regions in protein sequences. For example, APRs are frequently punctuated by charged or proline residues (labeled gate-keepers) [24], proteins with higher aggregation propensities have shorter half-lives in the cell [25], and mRNA expression levels in *E. coli* are lower for proteins with higher aggregation propensities [26]. A study linking protein evolution and aggregation discovered an overall decreasing trend for aggregation propensity among organisms with increasing complexity and longevity [30]. This implies that organisms have evolved by minimizing the aggregation propensity of their proteomes. Subsequently, it was determined that the aggregation propensity differences between prokaryotic and eukaryotic proteomes could be explained by differences in their number of IDPs, which have fewer APRs than ordered proteins do [27]. This contradictory finding, suggests that organisms may not have minimized the aggregation propensity of their proteomes during the course of evolution. In light of the above reports, there is a need to further investigate the various roles that aggregation prone regions have in protein structure and the concept that nature is actively minimizing the aggregation propensity of protein sequences.

This report presents findings from comprehensive analyses on multiple non-redundant datasets of protein sequences and structures (see Methods). Datasets were analyzed for aggregation propensity differences among monomeric proteins, IDPs, and randomized sequences with uniform and natural amino acid compositions. To assess if evolutionary selective pressure has minimized the aggregation propensity of protein sequences through APR disruption, average sequence identity among homologous proteins was compared to percent APR conservation among the same sequences. IDPs and monomeric proteins were used to evaluate the role that APRs have in promoting structural order and stabilizing interactions. Proximity of APRs to catalytic sites in enzyme structures was also investigated. Our findings suggest that proteins have evolved by optimizing their risk of aggregation for cellular environments through the overall minimization of aggregation prone regions and the conservation of those important for folding and function. In addition to promoting aggregation under conditions that destabilize proteins, APRs also stabilize protein structure and resist disorder, particularly, in structural areas that are important for protein function. Therefore, strategies employing site directed mutagenesis to improve protein solubility should carefully evaluate the contributions made by candidate APRs, targeted for disruption, towards protein structure and activity. The major findings from this work are summarized in Table 1.

**Table 1:** Summary of major findings reported in this article.

1.	The use of randomized sequence datasets has allowed us to deconstruct how changes in amino acid composition and patterning impact the aggregation propensity of a protein sequence.
2.	Multiple sequence alignments of homologues at the >50% sequence identity level show that APRs are often more conserved than other sequences regions are.
3.	Sequence analyses of intrinsically disordered proteins and monomeric proteins have provided direct evidence that APRs are more likely to contain ordered residues.
4.	APRs found in monomeric proteins often contribute more towards stabilizing a protein fold than the average contribution made by equal-length segments within the same protein.
5.	Catalytic residues frequently make close structural contact with APRs significantly more often than expected by random chance.
6.	APR disruption is an attractive rational protein engineering strategy for improving protein solubility. However, disruption of APRs, without knowledge of their contributions to protein structure and function, can lead to undesirable consequences, such as protein destabilization and/or loss-of-function.

## Results/Discussion

To assess the various roles that aggregation prone regions have in protein structure and the concept that evolution is actively minimizing the aggregation propensities of protein sequences, several non-redundant sequence datasets were generated (see Methods) that include: a library of experimentally proven amyloid-like fibril forming peptide sequences [31], [32] (Amylsegs); sequences and structures of 495 small (sequence length, 52–200 residues; average, 152±34) monomeric (both in crystal asymmetric units and in biological units) and non-homologous (sequence identities ≤30% in all against all alignments) proteins with high resolution crystal structures (R≤2.0 Å), (F495); 536 non-homologous IDP sequences obtained from the DisProt database [33] (IDP536); and 961 catalytic residues in 314 non-homologous protein chains (299 proteins) derived from a dataset of functional residues compiled by Xin et al. [34] (Cata). These datasets were supplemented with two random sequence datasets (R10000 and N10000) each with 10,000 amino acid sequences, 100 residues long. A uniform distribution of amino acids (5% for each amino acid) was used to generate random sequences for R10000. Random sequences in N10000 were generated from the amino acid distribution of naturally occurring protein sequences found in F495. Each protein sequence in F495 was also scrambled one hundred times to obtain a dataset, SF49500, which contains 49,500 sequences that have the same amino acid distribution, but not the same patterning as sequences in F495 (see Methods). The F495 dataset was also divided into two datasets, F1 and F2, of roughly equal number of sequences but significantly different amino acid compositions. Both F1 and F2 retain their natural protein sequence patterning but have different amino acid compositions. The results described in this report are organized into five sections. In the first section, evidence is presented that indicates the aggregation propensities of protein sequences have been minimized in comparison to random sequences with uniform or natural amino acid compositions. The second section describes an examination into whether evolution disrupts or conserves APRs in the sequences of homologous proteins. The third section compares the incidence of APRs in ordered versus disordered residues in proteins. This is followed by an evaluation of the contributions that all predicted APRs make towards stabilizing the proteins in which they exist compared to other protein segments of equal length from the same protein. The last section reports on the spatial proximity of catalytic residues and APRs to assess if APRs have a role in maintaining protein function.

### Protein sequence aggregation propensities are minimized

**Figure 1:**
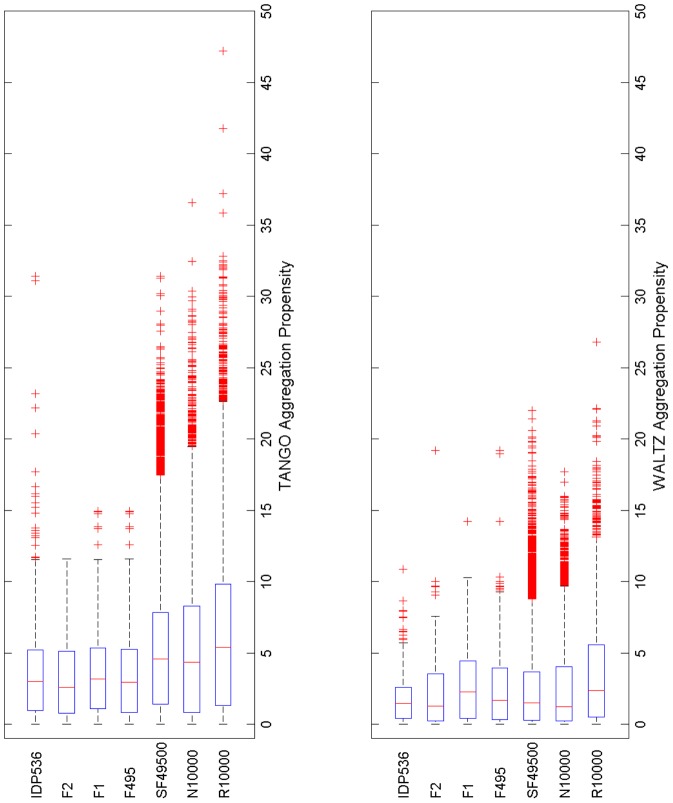
Box (blue rectangles) and whisker (dotted lines) plots for TANGO (top panel) and WALTZ (bottom panel) aggregation propensities (normalized TANGO/WALTZ aggregation scores) for different datasets used in this study is shown. The edges of the blue boxes represent inter-quartile ranges, 25 (Q1) and 75 (Q3) percentiles, and the medians are indicated by red vertical lines. In each plot, the whiskers represent limits of the distribution computed automatically, via tools available in MatLab2012a, and outlier points are represented as red colored +s. The axes are the same in all plots. This allows direct comparisons among plots for different datasets. It can be seen that TANGO aggregation propensities follow this order: R10000>N10000, SF49500>F495, F1, F2, IDP536. For WALTZ aggregation propensities, the trend appears to be R10000>N10000, SF49500, F495, F1, F2, IDP536. Also note that WALTZ predicted aggregation propensities tend to be lower than those of TANGO. These plots were made using MATLAB (www.mathworks.com).

Tables 2–4 present data on the various statistical measures of aggregation that were used in this study. Aggregation propensities were computed by normalizing total TANGO [11] and WALTZ [12] aggregation scores for protein sequences by their lengths. The average aggregation propensities for sequences of our various datasets are summarized in Table 2. For each dataset, the average length of protein sequences which contain at least one APR, total number of predicted APRs, average APR lengths and average proportion of APR residues in protein sequences are summarized in Table 3. The incidence of gate-keeper residues in APR flanking regions for sequences in R10000, N10000, SF49500, F495 and IDP536 datasets is presented in Table 4. Figure 1 shows box and whisker plots of the aggregation propensities for all datasets. These plots indicate an overlap among the aggregation propensity distributions for the various datasets. Overlaps are expected because the amino acid sequences in all datasets are composed of the same twenty types of naturally occurring amino acids found in proteins. Notwithstanding the overlaps, it can be seen that there are important differences among datasets in the inter-quartile ranges for both TANGO and WALTZ predicted aggregation propensities. To determine the statistical significance of aggregation propensity distribution differences among datasets, two sample t-tests were performed (See Methods) and the results are summarized in Table S1 of the Supplementary Material. In the following discussion, aggregation property averages (e.g. propensity or APR length, etc.) are compared among the various datasets. Standard deviations are provided along with averages.

**Table 2:** Average aggregation propensities of sequences in different datasets.

Dataset	Number of sequences	Average TANGO aggregation propensity*	Range of TANGO aggregation propensities*	Average WALTZ aggregation propensity*	Range of WALTZ aggregation propensities*
R10000	10,000	6.48±5.89	0–47.23	3.39±3.33	0–26.82
N10000	10,000	5.42±5.18	0–36.58	2.40±2.75	0–17.72
SF49500†	49,500	5.20±4.33	0–31.45	2.34±2.48	0–22.00
F495	495	3.41±2.98	0–14.94	2.52±2.70	0–19.20
All-α	66	3.24±3.03	0–13.88	2.53±3.06	0–18.98
All-β	53	3.59±2.87	0–9.28	2.78±3.42	0–19.20
α+β	88	3.58±2.92	0–12.59	2.67±2.70	0–14.23
α/β	43	4.95±2.72	0.41–10.68	2.37±2.12	0–9.67
Other	245	3.18±2.96	0–14.94	2.50±2.53	0–10.04
F1‡	252	3.66±3.16	0–14.94	2.84±2.67	0–14.23
F2‡	243	3.21±2.77	0–11.58	2.19±2.51	0–19.20
IDP536	536	3.85±4.05	0–31.42	1.74±1.60	0–10.89

* TANGO/WALTZ aggregation propensity of a sequence was computed as its total TANGO/WALTZ aggregation score normalized by the number of residues in the sequence.

† Dataset SF49500 was obtained by randomly scrambling each sequence in F495 a hundred times (see Methods for details).

‡ Datasets F1 and F2 were obtained by dividing F495 into two sets such that their amino acid compositions are significantly different (see Methods for details). Standard deviations (σ) are reported for all averages.

Standard error (SE) of the mean can be computed as σ/√(number of sequences) with 95% confidence intervals (average aggregation propensity <x> ± SE*1.96).

**Table 3:** Average properties of predicted APRs in different datasets.

Dataset	TANGO predicted APRs					WALTZ predicted APRs				
	Sequences with ≥1 APR (%)	Average Sequence length*	Total number of APRs	Average APR length (#)	Average proportion of APR residues (%)†	Sequences with ≥1 APR (%)	Average Sequence length*	Total number of APRs	Average APR length (#)	Average proportion of APR residues (%)†
R10000	68.9	100	10474	8.83±3.15	13.42±7.22	67.5	100	10292	6.14±0.67	9.36±4.66
N10000	62.6	100	8965	8.49±2.88	12.16±6.33	55.7	100	7698	6.12±0.63	8.46±3.92
SF49500	71.4	149±35	59878	8.48±2.85	10.27±5.40	63.6	148±35	51450	6.13±0.61	7.23±3.81
F495	60.4	152±34	452	7.47±1.99	7.85±4.08	63.2	147±35	538	6.13±0.68	7.71±3.91
All-α	63.6	139±37	62	7.79±2.73	8.91±4.89	59.1	138±38	61	6.25±1.15	7.71±4.02
All-β	64.2	150±38	47	7.60±2.06	7.63±3.17	66.0	146±33	59	6.15±0.55	7.52±4.43
α+β	63.6	153±31	82	7.23±1.82	7.20±2.81	67.0	144±34	97	6.21±0.85	7.60±3.48
α/β	79.1	167±22	66	7.11±1.42	8.35±3.53	72.1	165±22	58	6.07±0.41	7.02±3.78
Others	57.1	151±34	203	7.55±1.91	7.65±4.44	62.4	145±36	267	6.09±0.51	7.86±4.01
IDP536	75.2	763±562	1316	9.13±3.9	6.95±5.37	73.4	768±557	1372	6.09±0.54	4.71±2.70

* Average length of sequences that contain at least one TANGO/WALTZ predicted APRs.

† Average proportion of APR residues in the sequences contained in different datasets was computed as described in methods.

Sequences that do not contain APRs were excluded from both the average length and average proportion calculations. Standard deviations (σ) are reported for all averages. Standard error (SE) of the mean can be computed as σ/√(number of sequences; see Table 2) with 95% confidence intervals (average <x> ± SE*1.96).

**Table 4:** Average incidence of gate-keeper residues flanking APRs in different datasets*.

	R10000	N10000	SF49500	F495	All-α	All-β	α+β	α/β	Other	IDP536
TANGO predicted APRs										
Average # of Gate-keeper residues	2.2±1.1	2.4±1.1	2.5±1.1	2.6±1.2	2.6±1.3	2.6±1.2	2.5±1.3	2.8±1.1	2.5±1.2	2.4±1.1
Frequency at P_B_−1 (%)	53.6	58.9	58.3	57.1	59.7	44.7	57.3	65.2	57.1	56.8
Frequency at P_B_−2 (%)	30.4	35.1	35.8	42.0	43.5	44.7	41.5	50.0	40.4	37.8
Frequency at P_B_−3 (%)	26.2	30.2	31.3	31.6	32.3	34.0	26.8	33.3	32.5	28.9
Frequency at preceding flanking residues	36.7	41.4	41.8	43.6	45.2	41.1	41.9	49.5	43.3	41.2
Frequency at P_E_+1 (%)	44.0	50.3	52.0	51.1	61.3	53.2	53.7	39.4	51.7	45.1
Frequency at P_E_+2 (%)	34.8	37.3	38.2	41.2	45.2	48.9	36.6	51.5	36.0	38.5
Frequency at P_E_+3 (%)	27.4	30.8	31.8	33.8	22.6	31.9	35.4	45.4	34.5	32.2
Frequency at succeeding flanking residues	35.4	39.5	40.7	42.0	43.0	44.7	41.9	45.4	40.7	38.6
WALTZ predicted APRs										
Average # of Gate-keeper residues	1.7±1.1	1.9±1.1	1.9±1.1	2.0±1.1	2.0±1.0	2.1±1.2	2.2±1.1	2.2±1.2	1.9±1.1	2.0±1.2
Frequency at P_B_−1 (%)	29.2	33.4	33.6	37.9	42.6	39.0	45.4	46.6	31.1	33.6
Frequency at P_B−_2 (%)	26.7	31.4	31.7	32.9	37.7	39.0	32.0	34.5	30.0	34.8
Frequency at P_B_−3 (%)	25.7	29.6	30.4	30.5	24.6	30.5	34.0	36.2	29.6	32.4
Frequency at preceding flanking residues	27.2	31.5	31.9	33.8	35.0	36.2	37.1	39.1	30.2	33.6
Frequency at P_E_+1 (%)	29.9	34.2	34.8	35.3	37.7	32.2	32.0	39.7	36.0	37.3
Frequency at P_E_+2 (%)	27.5	32.0	31.9	34.0	27.9	40.7	42.3	31.0	32.2	32.7
Frequency at P_E_+3 (%)	26.7	29.7	30.8	32.2	27.9	32.2	33.0	36.2	32.2	32.1
Frequency at succeeding flanking residues	28.0	32.0	32.5	33.8	31.1	35.0	35.7	35.6	33.5	34.0

* Flanking positions preceding an APR are shown as P_B_−1, P_B_−2 and P_B_−3, where P_B_−1 is nearest to the APR. Similarly, flanking positions succeeding an APR are shown as P_E_+1, P_E_+2 and P_E_+3, where P_E_+1 is nearest to the APR.

Amino acid sequences in N10000 and SF49500 have a lower TANGO average aggregation propensity (i.e. average normalized aggregation score) than those in R10000 (Table 2). The average TANGO aggregation propensity for N10000 is 5.42±5.18, which is ∼16% lower than the corresponding value for R10000, 6.48±5.89. The average TANGO aggregation propensity for SF49500 is 5.20±4.33, almost 20% lower. Consistent with these averages, boxes representing inter-quartile ranges of TANGO aggregation propensities for N10000 and SF49500 are smaller (Figure 1) and the distributions of TANGO predicted aggregation propensities for N10000 and SF49500 are significantly different from those in R10000 at the 95% confidence level (Table S1). Interestingly, the percentage of sequences with at least one TANGO predicted APR in SF49500 is 71.4%, which is higher than that in N10000 (62.6%) or R10000 (68.9%). The number of APRs per sequence is also higher for SF49500 (59878 APRs/49500 sequences) = 1.21) than that for R10000 (10474/10000≈1.05) or N10000 (8965/10000≈0.90), (Table 3). However, the average length of APR containing sequences in SF49500 is 149±35, which is greater than that for R10000 and N10000 (length 100), and the average proportion of APR residues (see Methods, Equation 1) in SF49500 (10.3±5.4%) and N10000 (12.2±6.3%) is lower than those in R10000 (13.4±7.2%), (Table 3). These values clarify why the percentage of sequences with at least one TANGO predicted APR is greater in SF49500 than in R10000 but the average aggregation propensity is lower for SF49500 than in R10000.

The smaller average TANGO aggregation propensity of N10000 and SF49500 compared to R10000 is a consequence of their differences in amino acid composition. The amino acid distributions in N10000 and SF495000 are identical and significantly different from the amino acid distribution in R10000. The χ^2^ value for the amino acid distributions in R10000 versus N10000 (and SF49500) is 160.84. For 19 parameter distributions, χ^2^ values >43.82 reject the null hypothesis at the 99.9% confidence level (p-value<0.001) that the two amino acid distributions are the same [35]. N10000 and SF49500 contain both fewer aggregation-promoting residues and more gate-keeper residues than R10000. The total proportion of aggregation-promoting β-branched nonpolar (Ile and Val), aromatic (Phe, Tyr and Trp) and polar (Asn and Gln) residues is smaller in N10000 and SF49500 (29%) than in R10000 (35%) while the total proportion of gate-keeper charged and proline residues (Asp, Glu, Lys, Arg and Pro) is greater in N10000 and SF49500 (29.3%) than in R10000 (25%).

For WALTZ predicted APRs, the average aggregation propensities for N10000 and SF49500 are again smaller (2.40±2.75 and 2.34±2.48, respectively) than that for R10000 (3.39±3.33), (Table 2). WALTZ predicted APRs also constitute 7.2±3.8% of residues in SF49500 (8.5±3.9% in N10000) as compared to 9.4±4.7% in R10000, (Table 3). Note, only sequences that contain at least one APR were used in these calculations. WALTZ aggregation propensity box and whisker plot shows that the inter-quartile ranges for N10000 and SF49500 are smaller than the inter-quartile range for R10000 (Figure 1). The distribution of WALTZ aggregation propensities in R10000 is also significantly different from those in N10000 and SF49500 at 95% confidence level (Table S1). Other observations for N10000 and SF49500 versus R10000 show similar trends as in TANGO predictions (Tables 2 and 3). In summary, the above statistical measures have highlighted the importance of amino acid composition in explaining the average TANGO and WALTZ aggregation propensity differences between N10000 and SF49500 versus R10000.

Sequences of monomeric proteins in F495 have a lower average TANGO aggregation propensity than randomized sequences with natural amino acid composition (N10000 and SF49500), (Table 2). The amino acid sequences in N10000 and SF49500 lack the sequence patterning features of F495 due to randomization and scrambling (see Methods). The average TANGO aggregation propensity for F495 (3.41±2.98) is 34% lower than that for SF49500 (5.20±4.33) and 37% lower than that for N10000 (5.42±5.18)), (Table 2). Box and whisker plots of TANGO aggregation propensities indicate that the inter-quartile range for F495 is smaller and the third quartile (Q3) is shifted to lower values compared to SF49500 and N10000 (Figure 1). The distribution of TANGO aggregation propensities in F495 is also significantly different, at the 95% confidence level, from those in N10000 and SF49500 (Table S1). The aggregation propensity differences between F495 versus N10000 and SF49500 result from a similar, but broader, set of APR differences as those between N10000, SF49500 and R10000. Specifically, the percentage of sequences with at least one TANGO predicted APR decreases from 71.4% in SF49500 (62.6% in N10000) to 60.4% in F495, even though, the average length of APR containing sequences in F495 (152±34 residues) is similar in SF49500 (149±35), and longer than that in N10000 (100 residues), (Table 3). The average proportion of APR residues also decreases from 10.3±5.4% in SF49500 (12.2±6.3% in N10000) to 7.8±4.1% in F495 and the average APR length in F495 decreases by one residue from 8.48±2.85 in SF49500 (8.49±2.88 in N10000) to 7.47±1.99 in F495 (Table 3). The above analysis of TANGO predicted APRs reveals the importance of sequence patterning in explaining the average aggregation propensity differences between N10000 and SF49500 versus F495. Similar observations have been made by Chiti and coworkers *via* experiments on a 29 residue peptide from horse heart apomyoglobin. Sequence scrambled variants of the peptide showed substantial increases in aggregation propensity compared to the wild-type sequence due to clustering of amyloidogenic residues [36].

Aggregation propensity differences between F495 versus N10000 and SF49500 for WALTZ predicted APRs are dissimilar to those of TANGO predicted APRs (Tables 2 and 3, and Figure 1). The inter-quartile range for WALTZ predicted aggregation propensities in F495 is similar to the inter-quartile ranges for SF49500 and N10000 (Figure 1) and t-tests show that the distribution of WALTZ predicted aggregation propensities in F495 is statistically similar to SF49500 and N10000 distributions. The average WALTZ aggregation propensity for sequences in F495 (2.52±2.70) is comparable to that for sequences in N10000 (2.40±2.75) and SF49500 (2.34±2.48). The average APR lengths for WALTZ predicted APRs in SF49500 (6.13±0.61), N10000 (6.12±0.63) and F495 (6.13±0.68) are also the same (Table 3). The percentage of sequences that contain at least one WALTZ predicted APR in SF49500 and F495 are again similar at 63.6% and 63.2% respectively. The average proportion of APR residues in sequences of SF49500 (7.2±3.8%), N10000 (8.5±3.9%) and F495 (7.7±3.9%) are also similar (Table 3). From the observations above, scrambling sequences (SF49500) or producing random sequences with natural amino acid compositions (N10000) did not increase the number of WALTZ predicted APRs as it did for TANGO. WALTZ uses position-specific matrices that enable the program to predict APRs with fibril forming charged residues [12]. Consequently, sequence randomization may not have produced sequence patterns that WALTZ position-specific matrices have defined as aggregation prone.

After dividing F495 into different SCOP subclasses, the average aggregation propensity and other statistical measures of aggregation, such as proportion of sequences with at least one APR, number of APRs per sequence and average proportion of APR residues, remain similar to F495 for all subclasses (Tables 2 and 3), except in the α/β subclass. The α/β subclass contains only 43 (∼9%) proteins from F495. The average TANGO aggregation propensity of the α/β subclass (4.95±2.72) is higher than that for F495 (3.41±2.98), but the corresponding values for WALTZ aggregation propensities are similar (2.37±2.12 for α/β and 2.52±2.70 for all F495), (Table 2). A greater percentage of sequences in the α/β subclass contain one or more TANGO/WALTZ predicted APRs (TANGO, 79.1% for α/β subclass versus 60.4% for F495; WALTZ, 72.1% for α/β subclass versus 63.2% for F495) and the number of APRs per sequence is also higher for the α/β subclass (TANGO, 1.53 for α/β subclass versus 0.91 for F495; WALTZ, 1.35 for α/β subclass versus 1.09 for F495), (Tables 2 and 3). However, the average APR lengths in the α/β subclass are similar to that for all F495 protein sequences. Excluding the α/β subclass, the results in Tables 2 and 3 for the various SCOP classes suggest that differences in topology and secondary structure content alone do not produce average aggregation propensity changes that are similar to those observed after modifying sequence composition and patterning.

Average TANGO aggregation propensities are similar between IDP536 and F495 (3.85±4.05 for IDP536 and 3.41±2.98 for F495, Table 2). Box and whisker plots for IDP536 and F495 also show that these datasets have similar predicted aggregation propensities (Figure 1). The χ^2^-test on amino acid compositions of F495 and IDP536 yields a value of 20.4, thereby, accepting the null hypothesis that they have the same amino composition. The number of TANGO predicted APRs per sequence (2.46, Table 3) and the average APR length (9.13±3.90) is considerably larger in IDP536 compared to F495 (0.91 and 7.47±1.99, respectively). However, TANGO predicted APR differences between IDP536 and F495 are offset by longer sequence lengths in IDP536 (763±562), which results in a similar average proportion of APR residues (7.8±4.1% for F495 and 6.9±5.4% for IDP536, Table 3) and similar average TANGO aggregation propensities (Table 2). Longer APR lengths may result from lower sequence complexity among IDPs [33], [37]. For WALTZ, the average aggregation propensity (1.74±1.60) and average proportion of APR residues (4.7±2.7%) for IDP536 is lower than that for F495 (2.52±2.70 and 7.7±3.9%, respectively), but the average APR lengths (6.09±0.54 for IDP536 and 6.25±1.15 for F495) remain similar (Table 3). To assess if IDP536 and F495 actually have similar or different average aggregation propensities, the total proportion of ordered and disordered residues is needed for both datasets. This data is available for IDP536, but it is not for F495. Therefore, comparisons among ordered and disordered regions of protein sequences, instead of full length sequences from folded and intrinsically disordered proteins, are needed to better understand the relationship between aggregation and structural disorder (see section entitled *APRs have fewer disordered than ordered residues*).

An analysis of APR flanking residues among sequence datasets indicates that APRs are, on average, flanked more often by gate-keeper residues (Asp, Glu, Lys, Arg, or Pro) in F495, N10000, SF49500 and IDP536 datasets than in R10000 (Table 4). The average number of gate-keeper residues flanking TANGO predicted APRs in F495 is 2.6±1.2. Comparable values are observed for N10000 (2.4±1.1), SF495000 (2.5±1.1) and IDP536 (2.4±1.1). However for R10000, the corresponding value is lower at 2.2±1.1. For WALTZ predicted APRs, the average number of flanking gate-keeper residues in F495 is 2.0±1.1 which is comparable to averages for N10000 (1.9±1.1), SF49500 (1.9±1.1), and IDP536 (2.0±1.2). Gate-keeper residues flank WALTZ predicted APRs in R10000 less often at 1.7±1.1 (Table 4). Comparing the incidence of flanking gate-keeper residues in TANGO and WALTZ predicted APRs, gate-keeper residues flank WALTZ predicted APRs with lower frequencies (∼20–25% lower) than TANGO predicted APRs (Table 4). Charged gate-keeper residues oppose aggregation by keeping APRs solvated when they become exposed due to local flexibility or protein destabilization. However, WALTZ predicted APRs are more likely to contain charged or polar residues than TANGO predicted APRs due to the parameterization of WALTZ. As a result, the need to gate-keep WALTZ predicted APRs under normal conditions may be lower. Gate-keeper residues for all SCOP classes show similar trends as for F495, except for the α/β class which has slightly more gate-keeper residues (2.8±1.1 for TANGO predicted APRs and 2.2±1.1 for WALTZ predicted APRs, Table 4). Taken together, these observations suggest that the aggregation propensity of protein sequences has been minimized by increasing the number of gate-keeper residues in APR flanking regions, which is consistent with earlier studies [24], [38]. Gate-keeper residues occur with greater frequency in the flanking regions of APRs in F495, SF49500 and N10000 than in R10000 due to amino acid composition differences. However, N10000, SF49500 and F495 have identical amino acid compositions. Differences in the number of flanking gate-keepers residues between N10000/SF49500 versus F495 suggests that number of gate-keeper residues flanking APRs is also determined by sequence patterning as well as amino acid composition.

Selective pressure to reduce the burden of protein aggregation in biological organisms has minimized the aggregation propensities of protein sequences by directing changes to amino acid composition and patterning during protein evolution. Results presented in this section indicate that changes in sequence patterning *via* scrambling and randomization of protein sequences increases the TANGO predicted aggregation propensity more than do changes in amino acid composition. This is reflected in a greater difference in average aggregation propensity between F495/IDP536 and N10000/SF49500 than between SF49500/N10000 and R10000 (Tables 2 and 3) for TANGO, but not for WALTZ predicted APRs. To support the finding that changes to patterning impacts sequence aggregation propensity more than changes to amino acid composition, the F495 dataset was divided into two datasets (F1 and F2) that retain their natural protein sequence patterning but have significantly different amino acid compositions (see Methods). The average TANGO aggregation propensities for F1 (3.66±3.16) and F2 (3.21±2.77), and average WALTZ aggregation propensities for F1 (2.84±2.67) and F2 (2.19±2.51), are both similar to their respective values for F495 (TANGO 3.41±2.98; WALTZ 2.52±2.70), (Table 2). Furthermore, in the case of TANGO predicted APRs, the difference in average aggregation propensity between F1 and F2 is considerably less than it is between F495 and N10000/SF49500. Thus, changes to sequence patterning (F495 versus N10000) produce greater differences in average aggregation propensity than changes to amino composition do (F1 versus F2), at least for TANGO predicted APRs. Overall, the use of three different randomly generated sequence datasets, R10000, SF49500 and N10000, and three natural protein sequence datasets, F1, F2, F495, has allowed us separate the impact evolutionary selective pressure has had on amino acid composition and sequence patterning of protein sequences.

### APR conservation in protein homologues

A lack of APR conservation among homologous sequences is expected if protein evolution disfavors the tendency for proteins to aggregate [27], [30]. Here, the conservation of APRs among homologues of 9 proteins, selected from F495, was studied at two sequence identity cut-off values (see Methods). These 9 proteins contain ≥3 TANGO predicted APRs and ≥3 WALTZ predicted APRs or ≥3 Amylsegs, indicating these proteins have a high propensity to aggregate under suitable conditions. Table 5 shows APR and Amylseg conservation (Equation 11) among homologues of these 9 proteins at 80% and 50% sequence identity. Note these analyses did not include remote homologues (<30% sequence identity). The term ‘distant homologues’ used below refers to homologues that have at least 50% sequence identity with a parent sequence and the term ‘close homologues’ refers to homologues that have at least 80% sequence identity with a parent sequence. All homologues have the same fold as the parent protein. For the majority of these proteins, APR conservation levels are slightly lower than average sequence identity among close homologues. This observation is in agreement with that of Gromiha and coworkers [32] on protein sequence families containing highly homologous thermophilic and mesophilic proteins. However, when the sequence identity cut-off among homologues was dropped from 80% to 50%, the corresponding decrease in percent APR conservation is smaller. In other words, APRs are more conserved than average sequence identity among distant homologues that have at least 50% sequence identity to a parent sequence. Furthermore, amyloid-like fibril forming peptide segments (Amylsegs) in PDB entries 1KCQ (human gelsolin domain 2), 2D4F (human β-microglobulin), and 2VB1 (hen egg-white lysozyme), are highly conserved among their homologues at both sequence identities (50% and 80%). Overall, the data in Table 5 indicates that among close homologues, APRs are slightly less conserved than average sequence identity. A lack of APR conservation among closely related homologues suggests that evolution is continuing to disrupt aggregation prone sequence regions. On the other hand, among distantly related homologues, APRs are more conserved than average sequence identity, indicating that some of these regions may play a role in maintaining protein folds and activity.

**Table 5:** Conservation of APRs and Amylsegs among homologues of proteins contained in 9 selected PDB files†.

	80% sequence identity level!					50% sequence identity level#				
PDB entry and resolution*	Number of homologous sequences	Mean sequence identity (%)	Conserved TANGO APRs (%)*	Conserved WALTZ APRs (%)*	Conserved Amylsegs (%)	Number of homologous sequences	Mean sequence identity (%)	Conserved TANGO APRs (%)*	Conserved WALTZ APRs (%)*	Conserved Amylsegs (%)
1FUK,1.75 Å	29	86±4	82.8	87.3	No Amylsegs±	882	65±6	89.0	93.8	0±±
1JEO, 2.00 Å	2	90	33.3	40.0	No Amylsegs	6	68±19	33.3	36.4	No Amylsegs
1KCQ,1.65 Å	39	92±5	88.5	92.5	96.7	265	63±14	65.3	88.0	98.2
1OW1,1.80 Å	49	94±5	95.2	96.5	No Amylsegs	91	80±17	88.6	94.1	No Amylsegs
1SK7, 1.60 Å	3	99±1	75.0	75.0	No Amylsegs	27	58±15	80.9	83.5	No Amylsegs
1Z77, 2.00 Å	4	97±3	78.9	78.6	No Amylsegs	5	92±11	68.2	73.3	0
2D4F, 1.70 Å	68	89±4	78.9	No APRs##	98.4	158	77±13	72.7	64.9	98.8
2VB1, 0.65 Å	27	93±5	63.6	83.3	93.6	187	63±13	51.9	85.6	97.6
3NR5, 1.55 Å	31	95±1	89.5	94.0	No Amylsegs	78	81±14	83.9	91.2	No Amylsegs

† Instances where APR conservation is lower than mean sequence identity are shown in bold.

* Percent APR conservation (PAPR_conserved_) is described in eq.7 of the Methods section.

! All sequences have ≥80% sequence identify with the sequence of the protein contained in the PDB file (parent).

# All sequences have ≥50% sequence identify with the sequence of the protein contained in the PDB file (parent).

## Minimum window size was set to 6 residues in calculations (methods).

± No experimentally proven amyloid-like fibril forming peptide sequences contained in Amylsegs were detected.

±± An Amylseg was detected in one of the homologous sequences but it was not conserved.

### APRs have fewer disordered than ordered residues

Sequences from IDPs contain both ordered and disordered regions, which facilitates an investigation into the structural ordering of residues located in predicted APRs. Of the 232,321 residues in IDP536 sequences, 60,638 (26.1%) fall into regions annotated as intrinsically disordered by DisProt [33], the source database for IDP536. Amino acid residues not annotated as disordered regions were assumed to be ordered in this analysis. Therefore of the 232,321 residues in IDP536, 171,683 (73.9%) were assumed to be ordered. After predicting APRs in IDP536 using TANGO, it was found that 12,066 (5.2%) of the 232,321 IDP536 residues fall within one of 1,316 predicted APRs. Of the 12,066 APR residues, 1,327 (11% of the 12,066 APR residues) residues are disordered leaving the remaining 10,739 (89% of 12,066) residues as ordered. Using this data, a contingency table was prepared to categorize IDP536 residues as ordered and not part of an APR, or ordered and part of an APR (likewise for disordered residues, see Table 6). Performing a χ^2^ analysis on the contingency table rejects the null hypothesis (p<0.001) that the structural classification of an amino acid residue in IDP536, as ordered or disordered, is independent from the likelihood that the residue is part of an APR. Furthermore, the contingency table produces an odds ratio of 2.98∶1 indicating that an ordered residue is three times more likely to be part of an APR than a disordered one (see the tutorial by McHugh for more information on contingency tables and odds ratios [39]). WALTZ predicted APRs have comparable results (odds ratio 2.02∶1, see Table 6). Thus, these observations are independent of the APR prediction tool used. Similar to the results reported above, Schymkowitz and coworkers [21] have reported that ordered proteins have three times the number of APRs that IDPs do. Here, we report that APRs are three times more likely to contain ordered versus disordered residues which provides direct evidence that APRs are located in order forming regions. At a fundamental level, APRs are promoting local structural order in all conditions, irrespective of whether conditions stabilize or destabilize proteins.

**Table 6:** Incidence of intrinsically disordered residues in TANGO/WALTZ predicted APRs.

	Intrinsically disordered residues (#)	Ordered residues (#)	Total residues (#)
TANGO predicted APRs			
Residues within TANGO APRs	1327 (11%†, 2.2%±)	10739 (89%, 6.3%)	12066 (100%, 5.2%)
Residues outside TANGO APR	59311 (26.9%, 97.8%)	160944 (73.1%, 93.7%)	220255 (100%, 94.8%)
Total residues	60638 (26.1%, 100%)	171683 (73.9%, 100%)	232321 (100%, 100%)
Odds ratio	0.335	2.98	
Fisher's exact probability	0.00088 (<0.001)		
WALTZ predicted APRs			
Residues within WALTZ APRs	1268 (15.2%, 2.1%)	7096 (84.8%, 4.1%)	8364 (100%, 3.6%)
Residues outside WALTZ APRs	59370 (26.5%, 97.9%)	164587 (73.5%, 95.9%)	223957 (100%, 96.4%)
Total residues	60638 (26.1%, 100%)	171683 (73.9%, 100%)	232321 (100%, 100%)
Odds ratio	0.495	2.018	
Fisher's exact probability	0.0009 (<0.001)		

† Percentages are for numbers along the rows and add up to 100%.

± Percentages are for numbers along the columns and add up to 100%.

### APRs stabilize native protein folds

**Figure 2:**
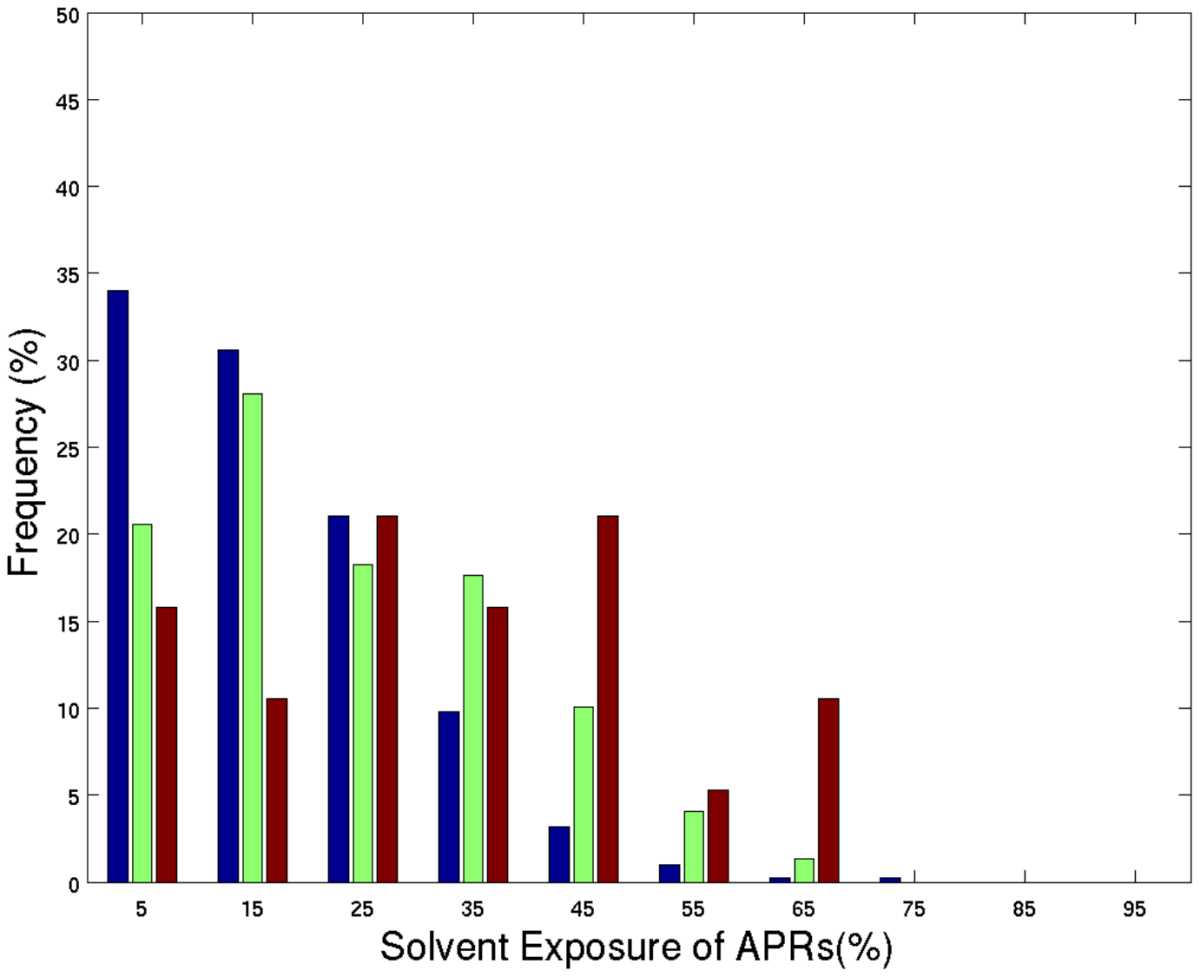
Solvent exposure presented in a bar plot for all 409 TANGO predicted APRs (blue bars), 516 WALTZ predicted APRs (green bars), and 19 Amylsegs (red bars) in the crystal structures of monomeric proteins from F495. X-axis shows the percent solvent accessible surface area and Y-axis indicates frequency of APRs and Amylsegs. On average, TANGO and WALTZ predicted APRs show lower solvent exposures compared to the Amylsegs.

The observation that APRs are more likely to contain ordered versus disordered residues suggests the need for an examination of APRs within globular protein structure. To perform the analysis, protein sequences from the F495 dataset, for which atomic coordinates are available, were used to investigate the secondary structure, solvent exposure, and relative solvent isolatedness of predicted APRs. Within F495 sequences regions with atomic coordinates, TANGO predicted 409 APRs, WALTZ predicted 516 APRs, and 19 Amylsegs of length ≥6 were found. Residues in these APRs occupy all three secondary conformational states (helix, coil, and strand) as measured by binning STRIDE [40] output (see Table S2 in Supplementary material). Overall, Amylsegs, TANGO, and WALTZ predicted APRs exist primarily in β-strands. This observation differs from the work of Doig and coworkers, who reported that amyloidogenic regions were often located in α-helical secondary structure [41]. Figure 2 compares the percent solvent exposure of TANGO and WALTZ predicted APRs and Amylsegs in F495 structures (Equations 2, 3 and 5). The average percent solvent exposure for TANGO predicted APRs is 16.9±12.2% (range 0–73%) and WALTZ predicted APRs is 23.3±14.9% (range 0–66%). On average, Amylsegs in F495 are more solvent exposed than TANGO or WALTZ predicted APRs (average percent solvent exposure 32.1±18.5% and range 1–62%). However, Amylsegs are longer than TANGO and WALTZ predicted APRs, with an average length of 11.5±10.4 residues. In comparison, the average APR lengths are 7.5±2.0 for TANGO predicted and 6.1±0.7 for WALTZ predicted APRs. Because proteins in F495 are small globular monomers with larger surface area to volume ratios, longer sequence segments are more likely to contain solvent exposed portions than smaller segments do. The low average percent solvent exposure of TANGO and WALTZ predicted APRs implies that these sequence regions are buried in their protein structures. Similar observations have been previously reported [22], [41].

Predicted APRs are expected to be buried in protein cores since sequence hydrophobicity is a differentiating physico-chemical property for APR prediction programs. Therefore, it is of interest to know whether predicted APRs are buried in protein cores beyond what is expected from the hydrophobicity of their constituent residues. To investigate this question, a measure called Burial Preference (BurPref) was devised (see Methods). The BurPref of an APR is the ratio of the observed APR solvent exposure to the expected APR solvent exposure calculated from the average surface areas of its constituent amino acid residues in F495 (Equations 2–4 and 6). If the BurPref of an APR is less than one, then the APR is more buried than expected and *vice versa*. The average BurPref for TANGO predicted APRs in F495 is 0.82±0.56 (range 0–4.15) and for WALTZ predicted APRs is 0.89±0.55 (range 0–3.69). This indicates that TANGO and WALTZ predicted APRs are, on average, more buried in the protein cores of F495 than expected. On the other hand, the average BurPref for the 19 Amylsegs is 0.97±0.47 (range 0.05–1.65), which indicates that these segments are not preferentially buried. BurPref values for TANGO and WALTZ predicted APRs suggests that sequence hydrophobicity alone cannot explain their degree of burial. Other factors are also important such as the role an APR has in providing protein stability or function. Overall, BurPref values for TANGO/WALTZ predicted APRs support the view [41] that the risk associated with aggregation prone sequence regions is minimized by their burial within protein structures. As a consequence, APR initiated aggregation may be limited to conditions in which the native structure is destabilized or in which APRs become exposed during co-translation or due to local protein flexibility.

Buried APRs can form multiple interactions within their protein structure and contribute towards stabilizing native folds. To quantify the contribution an APR makes towards the stability of a protein, solvent isolatedness (Iso) [42] was computed. Solvent isolatedness of a protein segment (such as an APR) measures the fraction (0–1) of its surface buried by the rest of the protein structure (Equations 3, 7, and 8). A large solvent isolatedness value for a protein segment indicates that most of its surface is interacting with the rest of protein. On the other hand, a small solvent isolatedness value for a protein segment indicates that most of its surface is solvent exposed and thus contributes few stabilizing protein interactions. The average solvent isolatedness (Iso) values for TANGO predicted APRs (0.83±0.12; range 0.27–1.0), WALTZ predicted APRs (0.77±0.15; range 0.34–1.0), and Amylsegs (0.68±0.19; range 0.38–0.99), all indicate that these sequence regions have multiple interactions within their parent protein structures and are thus mostly solvent protected.

**Figure 3:**
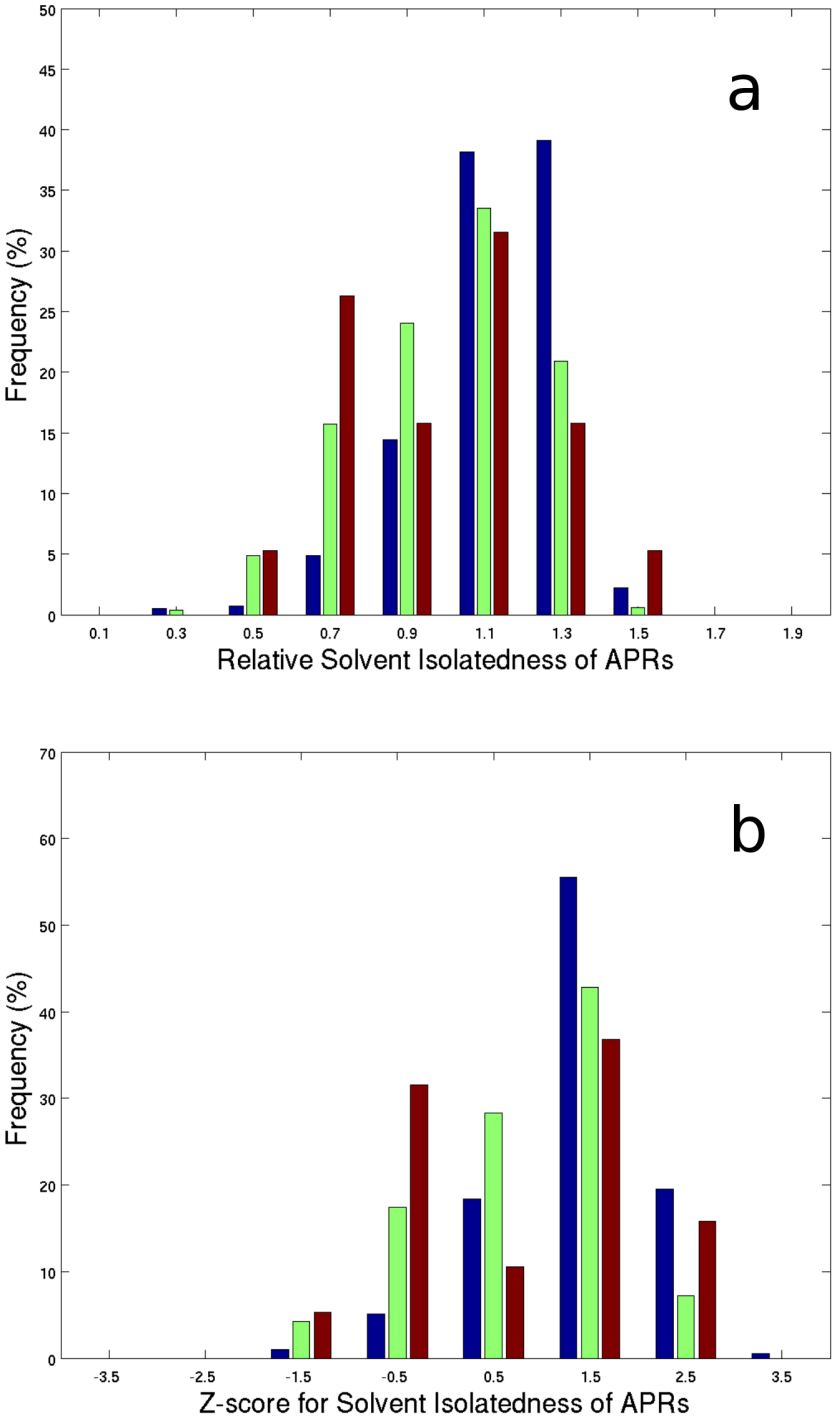
Solvent isolatedness of APRs and Amylsegs in the crystal structures of monomeric proteins from F495 is plotted. (a) Bar diagram showing the relative solvent isolatedness of TANGO predicted APRs (blue bars), WALTZ predicted APRs (green bars) and Amylsegs (red bars). X-axis shows relative solvent isolatedness and Y-axis indicates frequency. Most APRs and Amylsegs contribute more to the stability of native protein structures than expected from equal length segments from their parent proteins. (b) Bar diagram showing Z-scores for significance of solvent isolatedness values for TANGO predicted APRs (blue bars), WALTZ predicted APRs (green bars) and Amylsegs (red bars). X-axis shows the Z-scores and Y-axis indicates frequency. TANGO isolatedness Z-scores have an average of 1.44±0.76 indicating that APR isolatedness values are significant.

To assess if APR segments are more solvent isolated than expected, relative solvent isolatedness (RIso) was computed (Equations 7, 8 and 10). This quantity provides a measure of how much more an APR contributes to the stability of a protein than expected, based on the average solvent isolatedness of equal length segments from the same protein [42]. The relative solvent isolatedness values for TANGO and WALTZ predicted APRs and Amylsegs are greater than one which indicates that they are more solvent isolated than expected (TANGO average RIso 1.33±0.18, range 0.47–1.73; WALTZ average RIso 1.20±0.22, range 0.56–1.67; Amylsegs average 1.19±0.29, range 0.66–1.64), (Figure 3(a)). Therefore, TANGO and WALTZ predicted APRs, as well as Amylsegs, contribute towards protein stability more than expected. To assess the significance of APR solvent isolatedness values, Z-scores of solvent isolatedness were computed for predicted APRs and Amylsegs (Equations 7, 8 and 9). Although average Z-scores for WALTZ predicted APRs (0.88±0.95; range −1.9–2.8) and Amylsegs (0.78±1.14; range −1.2–2.4) are below one, the average Z-score for TANGO predicted APRs is 1.44±0.76 (range −1.9–3.3). Therefore, the contributions made by TANGO predicted APRs towards stabilizing the protein, in which they exist, are more than one sigma greater than the average contribution made by equal length segments (Figure 3(b)). Others have previously proposed that APRs make stabilizing interactions in native folds [25], [43]. However, this is the first report that TANGO/WALTZ predicted APRs and Amylsegs make more stabilizing interactions in native folds than, on average, those made by equal-length segments from the same protein.

### Catalytic residues are spatially proximal to APRs

Evidence that APRs stabilize native protein folds calls for an examination into whether functional sites are located within APRs or are structurally proximal to them. In this report, co-localization of catalytic sites and TANGO or WALTZ predicted APRs in crystal structures of enzymes is investigated using the Cata dataset which contains 961 catalytic residues from 314 non-homologous protein chains (299 enzymes). Catalytic residues form a subset of active site residues in enzymes. Thus, most enzymes contain few catalytic residues and the likelihood that these residues are located either within APRs, or near APRs, by random chance is low. Incidences of catalytic residues within TANGO/WALTZ predicted APRs and their flanking regions were estimated using equation 12 (see Methods). These estimated numbers were compared with the observed incidence of the catalytic residues within TANGO/WALTZ predicted APRs and their flanking regions. Ninety nine of the 961 catalytic residues (10.3%) were estimated to fall within TANGO predicted APRs and flanking regions and 57 of them (5.9%) were observed within these regions. Similarly, 103 out of the 961 catalytic residues (10.7%) were estimated and 69 (7.2%) were observed to fall within WALTZ predicted APRs and flanking regions. Therefore, the observed incidence of the catalytic residues within APRs and their flanking regions is lower than the estimates and it can be concluded that the catalytic residues are not usually found within these regions. This is expected given that most catalytic residues are charged or polar. On the other hand, catalytic residues do tend to be in structural contact with APRs (structural contacts are inferred when a catalytic residue heavy atom is within 4.5 Å from an APR residue heavy atom, see Methods). Of the 961 catalytic residues, 373 (38.8%) are in structural contact with at least one neighboring TANGO predicted APR residue. Similarly, 310 (32.3%) catalytic residues are in contact with at least one neighboring residue within WALTZ predicted APRs. Although, it has previously been observed that APRs and amyloidogenic regions can be located in protein-protein interfaces [22], [28], including antigen-antibody interfaces [23], this is the first report of structural proximity between APRs and catalytic residues to our knowledge.

**Figure 4:**
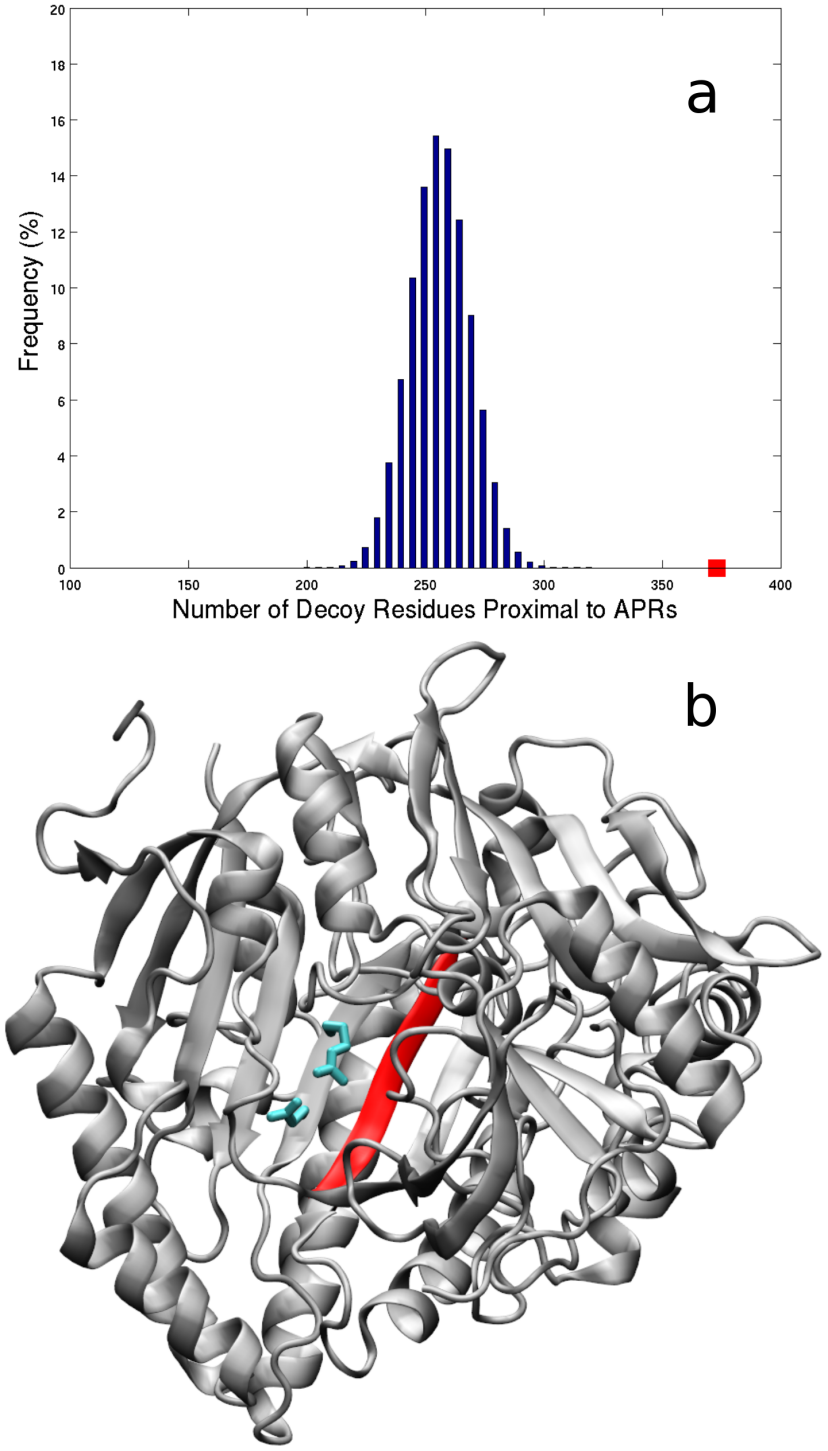
Catalytic residues are in contact with APRs more often than expected by random chance. (a) Histogram shows the number of residues (out of a maximum of 961) that are in contact with TANGO predicted APRs from 1,000,000 decoy catalytic residue lists (see Methods). X-axis shows the number of residues and Y-axis shows the frequency. There are 373 true catalytic residues in structural contact (using a distance cut-off of 4.5 Å) with the TANGO predicted APRs, shown by a red square along the X-axis. (b) Structural proximity between catalytic residues and a TANGO predicted APR in Cholesterol oxidase from *B. Sterolicum* is shown. Catalytic residues represented by cyan colored sticks are in contact with APRs represented by red colored ribbons. The image was made using chain A in PDB entry 1I19. Note, only structurally proximal catalytic residues and one APR are shown for simplicity.

To assess the significance of the observed structural proximity between APRs and catalytic residues, statistical simulations were performed by generating one million catalytic decoy lists. Each list contained the residue coordinates of 961 randomly chosen decoy catalytic residues from the atomic coordinates of protein chains in the Cata dataset. Randomly chosen decoy catalytic residues were selected for each true catalytic residue in Cata and were limited to any residue within the same protein structure as the true catalytic residue. Thus, if there are *X* number of true catalytic residues from protein *Y* in the Cata dataset, all decoy lists contained *X* number of decoy catalytic residues from the same protein *Y*. Using decoy catalytic lists enabled us to calculate an expected number of residues in structural contact with predicted APRs. This expected number was compared to the observed number of true catalytic residues in structural contact with predicted APRs. Figure 4(a) shows the distribution of the number of decoy catalytic residues which are in structural contact with TANGO predicted APR residues for all one million lists. The average number of decoy catalytic residues in contact with TANGO predicted APRs is 254±13. This yields a Z-score of 9.3 for the 373 of 961 true catalytic residues in the Cata dataset that are in structural contact with TANGO predicted APRs and suggests the number of true catalytic residues in contact with TANGO predicted APRs is highly significant. These calculations were repeated by varying the distance cut-off for inferring structural contacts (3.5 Å and 6.0 Å). The observed number of true catalytic residues in contact with TANGO predicted APRs is 278 and 461 at 3.5 Å and 6.0 Å, respectively. Statistical simulations to compute the expected number of randomly chosen decoy catalytic residues in contact with TANGO predicted APRs yield Z-scores of 10.1 at the 3.5 Å cut-off distance and 11.7 at the 6.0 Å cut-off distance for the number of true catalytic residues in contact with TANGO predicted APRs. Catalytic residues in the Cata dataset are also in close structural proximity to WALTZ predicted APRs, significantly more often than expected. There are 224, 310 and 405 catalytic residues in contact with WALTZ predicted APRs at cut-off distances of 3.5 Å (Z-score, 5.8), 4.5 Å (Z-score, 5.7) and 6.0 Å (Z-score, 8.5) respectively. To further probe our observation of catalytic residues in contact with APRs, an additional condition on the solvent exposure of randomly selected residues as decoy catalytic residues was imposed. Decoy catalytic residues were required to have a solvent exposure that was similar to the solvent exposure of their corresponding true catalytic residue (ASA value of each decoy catalytic residue must be within ±10% of the ASA value for the corresponding true catalytic residue). At the structural contact cut-off distance of 4.5 Å, Z-scores for catalytic residues in contact with TANGO and WALTZ predicted APRs are 5.9 and 3.1 respectively when ±10% ASA condition was imposed. As such, Z-scores decreased but remain statistically significant. Z-scores decreased when the ASA condition was imposed because the probability that a decoy catalytic residue is in contact with an APR increases when decoy catalytic residues are limited to the same solvent exposure as their true catalytic residues. Computing expected values for the number of residues in contact with APRs, using both ASA limitations and multiple distance cut-offs, has supported our finding that catalytic residues are in close structural contact with APRs significantly more often than expected by random chance.

To visualize the co-localization of APRs and catalytic residues in enzyme structures, an example from the Cata dataset is shown in Figure 4(b)). Cholesterol Oxidase from *B. Sterolicum* (PDB entry: 1I19, UniProt entry: Q7SID) is a 561 residue monomeric enzyme with covalently bound FAD that catalyzes oxidation and isomerization of steroids [44]. This enzyme contains three catalytic residues, namely, Glu 311, Glu 475 and Arg 477, and two TANGO identified APRs, 229-LTAVVW-234 and 511-VAIWLNVL-518. Two catalytic residues, Glu 475 and Arg 477, make several close contacts with the second APR (Figure 4(b)), which lies in the substrate binding domain [44]. Considering the large structural size of Cholesterol Oxidase, and the fact that is has only three catalytic residues and two TANGO predicted APRs, which are short in length, it is surprising to find its catalytic residues in structural contact with its APRs.

**Figure 5:**
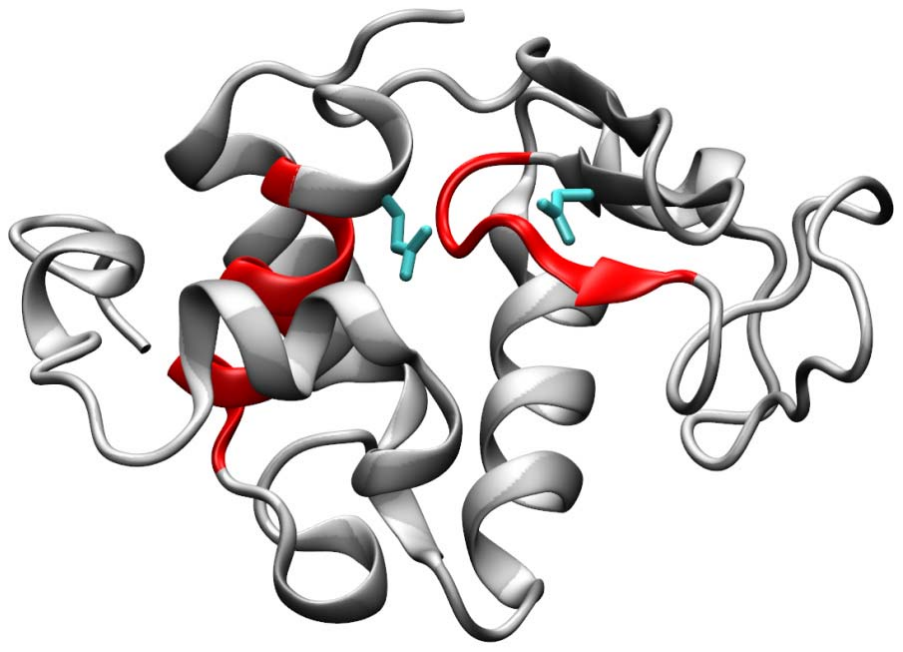
Catalytic residues that make structural contact (distance cut-off 4.5 Å) with Amylsegs of human lysozyme are shown. The human lysozyme (UniProtKB entry: P61626) contains two catalytic residues, Glu 35 and Asp 53. These residues are represented as cyan colored sticks and are in contact with Amylsegs (25-LANWMCLAKW-34 and 56-IFQINS-61) represented by red colored ribbons. The image was made using the PDB entry, 1IWT.

Are there examples of catalytic residues making structural contact with experimentally validated amyloid-fibril forming peptide segments, Amylsegs? To answer this question, the AmylSegs dataset was searched for peptide segments from enzymes. Amylsegs contain amyloid-fibril forming peptides derived from nine different enzymes. Six of these nine enzymes have been annotated in UniProtKB for catalytic residues and have at least one crystal structure deposited in the PDB (Table 7). Of these six, three enzymes (pancreatic ribonuclease A, hen egg white lysozyme and human lysozyme) have a catalytic residue that is in contact with at least one Amylseg residue. The criterion used here to identify a structural contact is the same as in analyses on the Cata dataset (distance cut-off of 4.5 Å for a pair of heavy atoms). Figure 5 shows an example of catalytic residues from human lysozyme, Glu 35 and Asp 53, which are in contact with its Amylseg regions. There are three peptides in the Amylsegs dataset from human lysozyme that have been experimentally shown to form amyloid fibrils. These are 5-RCELARTLKR-14, 25-LANWMCLAKW-34 and 56-IFQINS-61 [45]. Catalytic residue, Glu 35, lies immediately after the second peptide and makes contact with residues 30-CLAKW-34 as well as contact with residues 56-IFQ-58 from the third peptide. Catalytic residue Asp 53 also contacts the third peptide. Indeed, catalytic residues are making structural contact with experimentally validated amyloid-fibril forming peptide segments.

**Table 7:** Enzymes that contain Amylsegs in close structural contact with catalytic residues.

Protein, UniProt entry, PDB entry	Amyloidogenic region†	Component Amylsegs	Catalytic Residues#	Contacting Amylseg residues
Bovine Pancreatic Ribonuclease A, P61823, 1AFK	1-KETAAAKFERQHMDSSTSAASSSNY-25	1-KETAAAKFERQHMDSSTSAA-20	H12, H119	H12: F8-D14
	75-SYSTMSIT-82	15-SSTSAASSSNY-25		H119: F8, I107, A109
	101-QANKHIIVA-109	20-ASSSNY-25		
		75-SYSTMS-80		
		77-STMSIT-82		
		101-QANKHI-107		
		104-KHIIVA-109		
AChE, P22303, 2X8B	555-AEFHRWSSYMVHWK-568	555-AEFHRWSSYMVHWK-568	S203, E334, H447	Unknown*
Barnase, P00648, 1A2P	1-AQVINTFDGVADYLQTYHK-19	1-AQVINTFDGVADYLQTYHK-19	E72, H102	No
Hen Egg White Lysozyme, P00698, 2VB1	49-GSTDYGILQINSRWW CNDGRTPGSRNLCNIPCSALLSSDITASVNCAKKIVSDGNGMNA-107	49-GSTDYGILQINSRWWS-64	E35, D52	E35: I55- Q57
		57-QINSRWWCNDGRTPGSRNLCNIPCSALLSSDITASVNCAKKIVSDGNGMNA-107		D52: T51-G54, Q57-S60
		55-ILQINS-60		
Human Lysozyme, P61626, 1IWT	5-RCELARTLKR-14	5-RCELARTLKR-14	E35, D53	E35: C30-K34, I56-Q58
	25-LANWMCLAKW-34	25-LANWMCL61AKW-34		D53: Q58- S61
	56-IFQINS-61	56-IFQINS-61		
Human Prostatic Acid Phosphatase, P15309, 1ND6	216-GIHKQKEKSRLQGGVLVNEILNHMKRATQIPSYKKLIMY-254##	228-GGVLVN-233	H12, D258	No

† Amyloidogenic regions were identified by combining the overlapping peptides in the Amylsegs dataset. Each peptide has been shown in literature to form amyloid-like fibrils.

# The positions of catalytic residues, Amylsegs and Amyloidogenic regions follow PDB residue numbering.

* The PDB entry does not contain the coordinates for the amyloidogenic region in AChE.

## Amylsegs contain a hexapeptide sequence for human prostatic acid phosphatase.

Protein functional sites require optimal combinations of flexibility and stability to fulfill their biological purposes. Therefore, it is logical for catalytic residues, which require consistent and specific orientations, to be in contact with regions that promote local structural order and form stabilizing interactions. While this report has focused on catalytic residues, an interesting example of an Amylseg, 182-SFNNGDCFILD-192, containing a Ca^2+^ binding residue at D187 in human gelsolin has also been identified. The D187N mutation, which disrupts the metal binding site, leads to protein instability and amyloidosis in patients with a disease called familial amyloidosis-Finnish type [46]. Co-localization of metal catalyzed oxidation (MCO) sites and APRs have also been observed in the structures of therapeutic proteins where metal-ion leachates contribute towards drug product degradation [47].

### Protein solubility optimization strategy

The results presented above show that protein sequences have evolved by optimizing their risk of aggregation for cellular environments by both minimizing aggregation prone regions and conserving those that are important for folding and function. Outside the cell, protein aggregation is commonly encountered in the laboratory and is a major hurdle in successful development of protein based biotechnology products, such as biotherapeutics. For these applications, the aggregation propensity may need to be further reduced in order to enhance protein yields from cell cultures and to improve protein solubility, especially at high concentrations. Disruption of APRs *via* site directed mutagenesis is an attractive protein engineering strategy to improve protein solubility [13], [14], [23], [48]–[50]. Alternatively, ‘supercharging’ functional proteins with very high electrostatic surface charge has also been shown to improve protein solubility beyond levels normally observed for natural proteins [51]–[53]. Because APRs can also form part of HLA-DR binding T-cell immune epitopes [54], [55], disrupting APRs potentially leads to a lower risk of immunogenic reactions in patients receiving biotherapeutic drugs. Insights gained from this report caution that the contributions made by candidate APRs, targeted for disruption, towards protein stability and function should be considered when identifying sites that are suitable for rational mutagenesis. Disruption of APRs, without knowledge of their contributions, can lead to undesirable consequences, such as protein destabilization and/or loss-of-function. This work also complements our efforts to distinguish between ‘active’ and ‘inactive’ APRs in proteins [56], [57].

**Figure 6:**
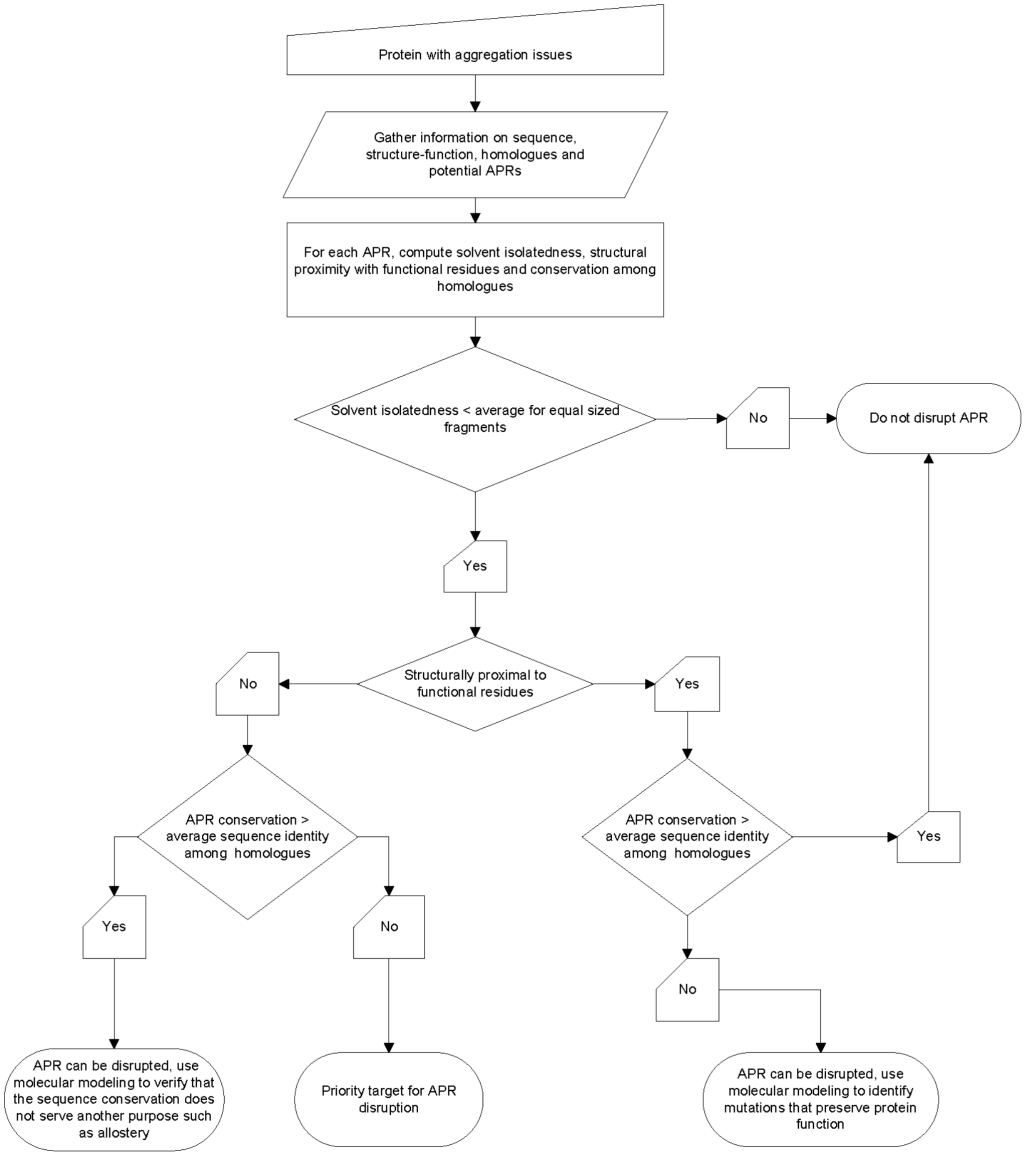
A general strategy for identifying mutation sites to improve protein solubility via disruption of APRs is shown. This strategy calls for an evaluation into the contributions made by candidate APRs towards the stability and function of a protein before deciding to disrupt it for the purpose of improving protein solubility. The following guidelines for rational disruption of APRs have emerged from this study. (i) If the candidate APR is buried in the protein core and contributes to the stability of the protein, then it should not be targeted for disruption. (ii) If the APR is located at or near the protein surface, is highly solvent exposed, not in contact with functional residues and is also less conserved than the average sequence identity among homologues to the parent sequence, then it is a priority target for disruption. Outside these two extremes, additional intermediate situations are identified where disruption of a candidate APR via point mutations can improve protein solubility without impacting its stability or function.

A broader implication of this research is that a general strategy for identifying mutation sites for improving solubility of a candidate protein can be proposed. This strategy is presented in Figure 6 and the major steps are described below. If a protein of biotechnological interest aggregates at higher than a desired level, the following information is needed to employ the strategy: protein sequence, three dimensional structure, homologues, potential cross-β motif forming APRs and functional sites. APR prediction programs often identify several potential APRs in the sequence of a protein. For each APR, its contribution towards protein stability should be evaluated. This can be done by computing solvent isolatedness for the APR and equal length segments from the same protein (Equation 8). If the APR has a high solvent isolatedness (low solvent exposure, buried in protein core) then it should not be a target for disruption. If the APR has a low solvent isolatedness (high solvent exposure, located at or near protein surface) then it is expected to make a smaller contribution to protein stability and can be marked for disruption depending upon the outcomes of the following tests. The protein structural region around the APR should be examined for contacting functional residues and for sequence conservation among homologues. If the APR contains residues that are in structural contact with functional residues and the APR is more conserved than average sequence identity among homologues, then it should not be targeted for disruption. If the APR is not in contact with functional residues and is less conserved than the average sequence identity among its homologues, then it is a priority target for disruption. If the APR is not structurally proximal to functional residues, but is more conserved than average sequence identity among its homologues, it can still be targeted for disruption after verifying that the conservation is not due other structure-function purposes such as allostery. If the APR is structurally proximal to functional residues, but is less conserved than average sequence identity among the homologues, it can still be targeted for disruption if molecular modeling can offer clues into potential sites and mutations that can be safely substituted. In this case, care should be taken to avoid disturbing the conformations of functional and contacting residues. This can be done by choosing a residue within the APR which is not in direct contact with functional residues, but whose mutation disrupts the APR.

## Methods

### Datasets used in this study

1. **F495** contains 495 non-redundant monomeric protein sequences, all of which have high resolution crystal structures available in the Protein Databank (PDB) [58]. F495 sequences were found by searching the PDB website (www.rcsb.org) with the following parameters: Experimental method, X-ray; Resolution, 2 Å or better; Free R-factor, ≤20%; Chain length, 50–200 residues; Sequence identity ≤30%; Asymmetric unit, 1 chain; Biologically active unit; 1 chain. The search yielded 522 structures. SEQRES records for these sequences were re-checked for redundancy (sequence identity ≤30%) by performing all-against-all sequence alignments in ClustalW [59]. 27 protein sequences were found redundant and discarded, reducing the data set to 495 proteins. Sequences of F495 were also assigned to SCOP fold classes [60] of which 66 were all-α, 53 all-β, 88 α+β, 43 α/β, and 245 were not classified or other.
2. **SF49500** contains 49,500 protein sequences obtained by scrambling each sequence in F495 a hundred times. Randomly selected amino acids in a parent sequence are assigned to new, randomly selected positions in a scrambled sequence until all amino acids have been reassigned. If a randomly selected position within a scrambled sequence is already occupied then new randomly selected positions are chosen until all unoccupied positions are filled. All scrambled sequences of a parent sequence have amino acid compositions and lengths that are identical to their parent sequence.
3. **F1 and F2** were obtained by dividing sequences from F495 using k-means clustering of individual sequence amino acid compositions. Clustering produced two new datasets with amino acid compositions that are significantly different from each other. The F1 dataset contains 252 (51%) sequences from F495 with amino acid composition, Ala, 6.2%; Cys, 1.8%; Asp, 5.9%; Glu, 8.8%; Phe, 4.0%; Gly, 6.5%; His, 2.7%; Ile, 6.1%; Lys, 8.9%; Leu, 8.1%; Met, 2.5%; Asn, 4.4%; Pro, 4.0%; Gln, 3.2%; Arg, 4.4%; Ser, 5.5%; Thr, 5.1%; Val, 7.2%; Trp, 1.2% and Tyr, 3.5%. The F2 dataset contains 243 (49%) sequences from F495 with amino acid composition, Ala, 9.1%; Cys, 2.1%; Asp, 5.8%; Glu, 6.2%; Phe, 3.3%; Gly, 7.8%; His, 2.7%; Ile, 4.6%; Lys, 4.4%; Leu, 9.2%; Met, 2.2%; Asn, 4.3%; Pro, 4.7%; Gln, 4.5%; Arg, 5.8%; Ser, 6.8%; Thr, 5.4%; Val, 6.5%; Trp, 1.4% and Tyr, 3.2%. The χ^2^ value for the two distributions is 89.01, indicating that they are significantly different from each other at 99.9% level of confidence.
4. **N10000** contains ten thousand, 100-residue long sequences that were randomly generated using the amino acid distribution obtained from sequences in F495: Ala, 7.5%; Cys, 1.8%; Asp, 5.8%; Glu, 7.5%; Phe, 3.8%; Gly, 7.1%; His, 2.8%; Ile, 5.4%; Lys, 6.5%; Leu, 8.7%; Met, 2.3%; Asn, 4.3%; Pro, 4.4%; Gln, 3.8%; Arg, 5.1%; Ser, 6.2%; Thr, 5.3%; Val, 6.9%; Trp, 1.4% and Tyr, 3.4%.
5. **R10000** contains ten thousand, 100-residue long sequences that were randomly generated with a uniform distribution of 5% for each amino acid.
6. **IDP536** contains 536 non-redundant (sequence identity ≤30%) protein sequences which have at least one intrinsically disordered region. IDP536 sequences were taken from the DisProt database V6 (www.disprot.org) [33]. All IDP536 sequence regions that are not annotated as disordered were annotated as ordered. Sequences from IDP536 have the following amino acid composition: Ala, 7.5%; Cys, 1.4%; Asp, 5.8%; Glu, 7.7%; Phe, 3.3%; Gly, 6.9%; His, 2.2%; Ile, 4.6%; Lys, 6.5%; Leu, 8.5%; Met, 2.3%; Asn, 4.3%; Pro, 5.8%; Gln, 4.9%; Arg, 5.2%; Ser, 7.9%; Thr, 5.6%; Val, 6.0%; Trp, 1.0% and Tyr, 2.7%.
7. **Cata** is adapted from Xin and Radivojac [34] and contains annotated catalytic residues from structures available in the PDB. Sequences from proteins in Cata were also checked for redundancy (sequence identity ≤30%) yielding a final dataset of 961 catalytic residues in 299 enzymes (314 unique polypeptide chains).
8. **Amylsegs** contains 517 non-identical, experimentally verified amyloid forming peptide sequences. Peptides in Amylsegs are part of full length amyloidogenic protein sequences. Amylsegs dataset curation has been described elsewhere [31], [32].

### APR and gate-keeper prediction

TANGO [11] and WALTZ [12] were used to predict APRs in all sequence datasets. Both programs have been extensively validated using independent testing sets and found to be highly accurate. The following options were used as input parameters for both programs: Temperature, 298K; pH, 7.0; Ionic Strength, 150 mM; Concentration 1 mM; TANGO/WALTZ aggregation, ≥10%; Minimum window size, 6; Flanking residues, 3.

### Statistical measures of aggregation and APRs

The outputs from TANGO and WALTZ yield data on the total sequence aggregation score and sequence length along with position, length, sequence and flanking residues for each predicted APR. The total aggregation score for each sequence was normalized by the length of the sequence to obtain its aggregation propensity. The proportion of APR residues (APRprop(%)) in a sequence was also computed as follows:
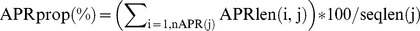
(1)APRlen(i, j) is the length of the i^th^ APR in the j^th^ sequence, nAPR(j) is the number of APRs in the j^th^ sequence, seqlen(j) is the number of residues in the j^th^ sequence. Averages and standard deviations were computed for both aggregation propensities and proportional APR residues. These results are reported in Tables 2 and 3.

Flanking residue positions that precede (P_B_−1, P_B_−2, P_B_−3) and succeed (P_E_+1, P_E_+2, P_E_+3) each APR were searched for the presence of gate-keeper residues (Asp, Glu, Lys, Arg and Pro) [24], [25] and their frequencies were computed at each of these positions. These results are presented in Table 4.

### Solvent exposure, burial preference and isolatedness of APRs

Atomic coordinates from F495 proteins were submitted to STRIDE [40] to obtain secondary structure information and solvent accessible surface areas (ASA) for all residues within their three dimensional context. Average ASA values for each of the twenty amino acids in the F495 dataset were obtained by summing the ASAs of amino acid, *k*, in all proteins and dividing the sum by the number of *k* amino acids in F495, where *k* runs from 1 to 20.
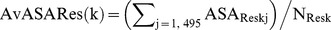
(2)AvASA_Resk_ is the average ASA for amino acid of type *k*. ASA_Reskj_ is the ASA of each amino acid residue of type *k* in protein *j* and N_Resk_ is the number of amino acid residues of type *k* in F495 dataset. ASA values for individual residues of APRs were summed to obtain observed solvent accessible surface areas (SASAobs) for each predicted APR.
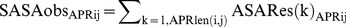
(3)APRij is the i^th^ APR in the j^th^ protein from F495. SASAobs_APRij_ is the observed SASA of APRij. ASARes(k)_APRij_ is the ASA of an individual residue, *k*, in APRij. The summation runs over the length of APRij, APRlen(i,j). Expected ASA values for each APR (SASAexp) were computed by summing the average ASA values from F495 for each of the constituent amino acid residues.
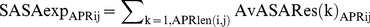
(4)SASAexp_APRij_ is the expected SASA of APRij. AvASARes(k)_APRij_ is the average ASA of residue type *k* in APRij, computed using Equation 2. The total surface area (TotSA) for an APR outside of its three-dimensional context was computed by submitting atomic coordinates, only from APR segments, to STRIDE. Note that in both calculations, APRs have identical conformations. TotSA and SASA values for each APR were used to compute percent solvent accessibility (SolvAcc) of the APR, burial preference (BurPref) for an APR, and solvent isolatedness (Iso) of an APR from solvent [42] as follows:
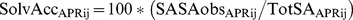
(5)

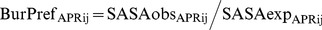
(6)

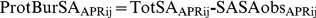
(7)

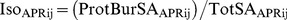
(8)SolvAcc_APRij_ is the percent solvent accessibility of APRij. SASAobs_APRij_ is the solvent accessible surface area of APRij within its three-dimensional context. TotSA_APRij_ is the total surface area of the APR outside of the three-dimensional context of protein *j*. SASAexp_APRij_ is the expected solvent accessible surface area of APRij computed from average ASA values for its constituent amino acids in F495. BurPref_APRij_ is a ratio of the observed to expected solvent accessible surface area for APRij. It indicates the preferential burial of APRij in protein cores. If BurPref_APRij_ is below one, APRij is more buried than expected from the average burial of its constituent residues. If BurPref_APRij_ is above one, APRij is more solvent exposed than expected from the average solvent exposure of its constituent residues. ProtBurSA_APRij_ is the surface area of APRij that is buried by the rest of the protein *j*. Iso_APRij_ values can be interpreted as the contribution APRij makes towards the stability of protein *j*. To evaluate the significance of this contribution, Iso_APRij_ values were also computed for all segments the equal length as APR, *i* in protein, *j*. Each segment was obtained by sliding a window the equal length as APR, *i* over the structure of protein, *j* one residue at a time. Average (<Iso_ij_>) and standard deviation (σIso_ij_) values for segments within protein *j* were used to compute Z-scores (Z-score (Iso_APRij_)) and relative values (RIso_APRij_) for solvent isolatedness of APRij using the following equations:
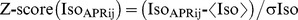
(9)

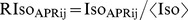
(10)


### APR and Amylsegs conservation

Nine selected monomeric proteins from F495 were used for sequence conservation analyses. These 9 proteins contain ≥3 TANGO predicted APRs and ≥3 Waltz predicted APRs or ≥3 Amylsegs, indicating these proteins have a high propensity to aggregate under suitable conditions. PDB entries for these proteins are 1FUK (C-terminal domain of yeast initiation factor 4A), 1JEO (Hypothetical protein MJ1247 from *Methanococcus jannaschii*), 1KCQ (Human Gelsolin Domain 2), 1OW1 (SPOC domain of human transcriptional factor SHARP), 1SK7 (Hypothetical protein pa-HO from *Pseudomonas aeruginosa*), 1Z77 (Transcriptional regulator (tetR family) from *Thermotoga maritima*), 2D4F (Human β-microglobulin), 2VB1 (Hen Egg White Lysozyme) and 3NR5 (Human RNA polymerase III transcription repressor Maf1). Note that Hen egg-white lysozyme, human β-microglobulin and human gelsolin are well studied amyloidogenic proteins [61]–[63].

Sequence conservation analyses for the above mentioned proteins were performed at two arbitrarily chosen levels of sequence identity, 80% and 50%. The procedure for selecting homologues at the 80% level is described below. Sequences of the nine proteins (query sequences) were searched for homologues in the UniProtKB database (www.uniprot.org) [64], [65] using blastp and all default options. For each query, hit sequences with ≥80% sequence identity were selected, provided that homologous regions of hit sequences covered the query sequence completely. Since hits to query sequences were sometimes longer than the length of the query sequence, only portions of hit sequences that aligned with the query sequence were taken for conservation analysis. All the retrieved homologous sequences were re-aligned using ClustalW [59]. For each query sequence, the ClustalW input file included the sequence from the PDB file as the first sequence. Any sequence with a ClustalW alignment score of <80 to the first sequence was deleted from the alignment. Sequences with alignment scores of 100 to the first sequence were also removed. All the above steps were repeated to obtain homologous sequences with ≥50% sequence identity to selected PDB files.

An APR was labeled as ‘conserved’ between two homologous sequences, if the APR has the same sequence in both homologues. Percent APR conservation in a multiple sequence alignment was computed using the following formula:
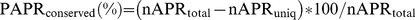
(11)PAPR_conserved_ is the proportion of conserved APRs in an alignment, nAPR_total_ is the total number of APRs for all the sequences in the alignment and nAPR_uniq_ is the number of unique APRs (non-identical sequence) over all sequences in the alignment. These calculations were performed for both TANGO and WALTZ predicted APRs. Analogous calculations were also performed for all peptide sequences from Amylsegs that were detected in the 9 proteins and their homologues.

### Incidence of catalytic residues in APRs and flanking regions

Number of the catalytic residues from the Cata dataset that fall within the TANGO/WALTZ predicted APRs and their flanking regions were estimated using the following equation:
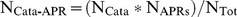
(12)N_Cata-APRs_ is the estimated number of catalytic residues that fall within TANGO/WALTZ predicted APRs and their flanking regions. Three residues preceding and three residues succeeding an APR are considered as its flanking regions. N_Cata_ is the number of catalytic residues in the Cata dataset. This number is 961. N_APRs_ is the number of residues in APRs and their flanking regions predicted using TANGO and WALTZ in the sequences of the 299 enzymes (314 Chains) in the Cata dataset. For 823 TANGO predicted APRs and their flanking regions, N_APRs_ is 11,414 (6498 residues in TANGO APRs plus 4916 residues in the flanking regions). N_APRs_ is 11,856 for 982 WALTZ predicted APRs and their flanking regions (5988 residues in the APRs and 5868 residues in the flanking regions). N_Tot_ is 110,334, the total number of residues in the sequences of the 299 enzymes.

### Structural proximity of catalytic residues to APRs

TANGO and WALTZ predicted APRs were also mapped onto protein structures from the Cata dataset to search for structural contacts made by catalytic residues to residues in predicted APRs. A catalytic residue is considered to be in structural contact with an APR, if at least one of its heavy atoms is within 4.5 Å from a heavy atom in any residue that falls within an APR. The choice of a 4.5 Å cut-off is arbitrary but was used here because it is common in the literature [66]–[68]. Catalytic residues in structural contact with APRs residues were counted for both TANGO and WALTZ predicted APRs.

To assess the significance of the observed structural proximity between APRs and catalytic residues, statistical simulations were performed by generating one million decoy catalytic lists. Each list contained the residue coordinates of 961 randomly chosen decoy catalytic residues from the atomic coordinates of protein chains in the Cata dataset. Randomly chosen decoy catalytic residues were selected for each true catalytic residue in Cata and were limited to any residue within the same protein structure as the true catalytic residue. For each of the 1,000,000 randomly generated lists, the number of residues making structural contact with APR residues (APR contacting) was computed again in the same way as for the Cata dataset.

The number of APR contacting residues was counted for each random list to generate a distribution of expected APR contacting residues. This distribution was also used to compute Z-scores for the incidence of APR contacting residues in the Cata dataset in the same way as the Z-score for solvent isolatedness was calculated (Eq. 9). The analogous calculations were also performed using the contact distance cut-off values of 3.5 Å and 6.0 Å.

To further probe our observation of catalytic residues in contact with APRs, a restriction on the solvent exposure of randomly selected residues as catalytic decoys was imposed. Decoy catalytic residues were required to have a solvent exposure that was similar to their corresponding true catalytic residue (ASA value of each decoy must be within ±10% of ASA of true catalytic residue). For each of the 1,000,000 randomly generated lists, the number of residues making structural contact with APR residues (APR contacting) was computed again in the same way as for the Cata dataset.

## Supporting Information

Table S1 Calculated p-values for two sample t-tests on the distributions of aggregation propensities for sequences contained in various datasets used in this work. ^†^p-values<0.05 indicate that the two datasets have significantly different distributions of aggregation propensities (yellow) at the 95% level of confidence. Datasets that have similar distributions of aggregation propensities are colored blue (p-value>0.05). The p-values for TANGO predicted aggregation propensities are shown in the upper triangle and the p-values in the lower triangle are for WALTZ predictions. The two-sample t-tests were performed using MATLAB (www.mathworks.com).
(DOCX)

Table S2 Secondary structure assignments for residues in TANGO/WALTZ predicted APRs and Amylsegs.
(DOCX)
