# Supplementary material for: On the Role of Aggregation Prone Regions in Protein Evolution, Stability, and Enzymatic Catalysis: Insights from Diverse Analyses
Source: PLoS Comput Biol. 2013 Oct 17;9(10):e1003291. doi: 10.1371/journal.pcbi.1003291 (PMC3798281; doi:10.1371/journal.pcbi.1003291)
Supplement: Table S1 — Calculated p-values for two sample t-tests on the distributions of aggregation propensities for sequences contained in various datasets used in this work. †p-values<0.05 indicate that the two datasets have significantly different distributions of aggregation propensities (yellow) at the 95% level of confidence. Datasets that have similar distributions of aggregation propensities are colored blue (p-value>0.05). The p-values for TANGO predicted aggregation propensities are shown in the upper triangle and the p-values in the lower triangle are for WALTZ predictions. The two-sample t-tests were performed using MATLAB (www.mathworks.com). (DOCX) [file pcbi.1003291.s001.docx]

**Table S1.** Calculated p-values for two sample t-tests on the distributions of aggregation propensities for sequences contained in various datasets used in this work^†^

|  | R10000 | N10000 | SF49500 | F495 | F1 | F2 | IDP536 |  |
| --- | --- | --- | --- | --- | --- | --- | --- | --- |
| R10000 |  | 0 | 0 | 0 | 0 | 0 | 0 | TANGO |
| N10000 | 0 |  | 0 | 0 | 0 | 0 | 0 |  |
| SF49500 | 0 | 0.017 |  | 0 | 0 | 0 | 0 |  |
| F495 | 0 | 0.362 | 0.105 |  | 0.286 | 0.376 | 0.051 |  |
| F1 | 0.009 | 0.014 | 0.001 | 0.127 |  | 0.09 | 0.524 |  |
| F2 | 0 | 0.231 | 0.355 | 0.112 | 0.006 |  | 0.026 |  |
| IDP536 | 0 | 0 | 0 | 0 | 0 | 0.003 |  |  |
|  | WALTZ | | | | | | |  |

^†^p-values < 0.05 indicate that the two datasets have significantly different distributions of aggregation propensities (yellow) at the 95% level of confidence. Datasets that have similar distributions of aggregation propensities are colored blue (p-value >0.05). The p-values for TANGO predicted aggregation propensities are shown in the upper triangle and the p-values in the lower triangle are for WALTZ predictions. The two-sample t-tests were performed using MATLAB (www.mathworks.com).

|  | | | |
| --- | --- | --- | --- |
|  |  | | |
|  |  |  |  |
|  |  |  |  |
|  |  |  |  |
|  |  |  |  |
|  |  |  |  |
