# Supplementary material for: On the Role of Aggregation Prone Regions in Protein Evolution, Stability, and Enzymatic Catalysis: Insights from Diverse Analyses
Source: PLoS Comput Biol. 2013 Oct 17;9(10):e1003291. doi: 10.1371/journal.pcbi.1003291 (PMC3798281; doi:10.1371/journal.pcbi.1003291)
Supplement: Table S2 — Secondary structure assignments for residues in TANGO/WALTZ predicted APRs and Amylsegs. (DOCX) [file pcbi.1003291.s002.docx]

| **Table S2.** Secondary structure assignments for residues in TANGO/WALTZ predicted APRs and Amylsegs | | | |
| --- | --- | --- | --- |
| Segment type | Residue conformation type | | |
|  | Helix (%)  (Mean, range) | Coil (%)  (Mean, range) | Strand (%)  (Mean, range) |
|  |  |  |  |
| 409 TANGO predicted APRs | 35.5±45.3, 0 - 100 | 13.2±17.8, 0 - 100 | 51.3±41.7, 0 - 100 |
| 516 WALTZ predicted APRs | 32.6±44.3, 0 - 100 | 18.3±23.9, 0 - 100 | 49.1±41.2, 0 - 100 |
| 19 Amylsegs | 5.4±14.7, 0 – 50 | 39.8±20.1, 0 - 80 | 54.8±25.2, 0 - 100 |
